# Multi-omics Mendelian randomization integrating GWAS, eQTL and pQTL data revealed GSTM4 as a potential drug target for migraine

**DOI:** 10.1186/s10194-024-01828-w

**Published:** 2024-07-22

**Authors:** Xinyue Sun, Bohong Chen, Yi Qi, Meng Wei, Wanying Chen, Xiaoyu Wu, Qingfan Wang, Jiahao Li, Xiangyu Lei, Guogang Luo

**Affiliations:** 1 Department of Neurology, The First Affiliated Hospital of Xi’an Jiaotong University, Xi’an, Shaanxi 710061 China; 2 Department of Urology, The First Affiliated Hospital of Xi’an Jiaotong University, Xi’an, Shaanxi 710061 China

**Keywords:** Migraine, Drug target, GSTM4, Mendelian randomization, Summary-data-based MR, Colocalization analyses

## Abstract

**Introduction:**

Migraine, as a complex neurological disease, brings heavy burden to patients and society. Despite the availability of established therapies, existing medications have limited efficacy. Thus, we aimed to find the drug targets that improve the prognosis of migraine.

**Method:**

We used Mendelian Randomization (MR) and Summary-data-based MR (SMR) analyses to study possible drug targets of migraine by summary statistics from FinnGen cohorts (nCase = 44,616, nControl = 367,565), with further replication in UK Biobank (nCase = 26,052, nControl = 487,214). Genetic instruments were obtained from eQTLGen and UKB-PPP to verify the drug targets at the gene expression and protein levels. The additional analyses including Bayesian co-localization, the heterogeneity in dependent instruments(HEIDI), Linkage Disequilibrium Score(LDSC), bidirectional MR, multivariate MR(MVMR), heterogeneity test, horizontal pleiotropy test, and Steiger filtering were implemented to consolidate the findings further. Lastly, drug prediction analysis and phenome-wide association study(PheWAS) were employed to imply the possibility of drug targets for future clinical applications.

**Result:**

The MR analysis of eQTL data showed that four drug targets (PROCR, GSTM4, SLC4A1, and TNFRSF10A) were significantly associated with migraine risk in both the FinnGen and UK Biobank cohorts. However, only GSTM4 exhibited consistent effect directions across the two outcomes(Discovery cohort: OR(95%CI) = 0.94(0.93–0.96); *p =* 2.70e − 10; Replication cohort: OR(95%CI) = 0.93(0.91–0.94); *p =* 4.21e − 17). Furthermore, GSTM4 passed the SMR at *p* < 0.05 and HEIDI test at *p* > 0.05 at both the gene expression and protein levels. The protein-level MR analysis revealed a strong correlation between genetically predicted GSTM4 with a lower incidence of migraine and its subtypes(Overall migraine: OR(95%CI) = 0.91(0.87–0.95); *p =* 6.98e-05; Migraine with aura(MA): OR(95%CI) = 0.90(0.85–0.96); *p =* 2.54e-03; Migraine without aura(MO): OR(95%CI) = 0.90(0.83–0.96); *p =* 2.87e-03), indicating a strong co-localization relationship (PPH4 = 0.86). Further analyses provided additional validation for the possibility of GSTM4 as a migraine treatment target.

**Conclusion:**

This study identifies GSTM4 as a potential druggable gene and promising therapeutic target for migraine.

**Supplementary Information:**

The online version contains supplementary material available at 10.1186/s10194-024-01828-w.

## Background

Migraine is defined as moderate to severe headache that lasts from 4 to 72 h, with reversible neurological and systemic symptoms [1]. Over 1 billion individuals worldwide are directly affected by this chronic, frequently lifelong illness [2, 3]. The one-year prevalence in people is estimated to be approximately 15%, with a female to male ratio of 3:1 [4]. Migraine with conditions including stroke [5], epilepsy [6], depression [7], and anxiety [8] frequently coexist.

Treatment of migraine includes acute treatment and prevention treatment. NSAIDs are used as an acute migraine therapy for mild to moderate pain, and triptans are used for moderate to severe pain [1]. Preventative therapies can reduce migraine attack frequency. The drugs targeting the CGRP pathway are used for both acute treatment and migraine attack prevention [9]. However, many current migraine treatments are ineffective. The non-responder rate of novel anti-migraine therapeutic agents targeting the CGRP system is even approximately 30% [10, 11], and its efficacy and long-term safety in patients with recurrent attacks and frequent administration have yet to be proved [12]. Thus, consideration of a wider spectrum of drug targets is important for patients with migraine.

Drug development requires the precise identification of therapeutic candidates for a certain disease and the confirmation of their effect on the progression of disease. That is, however, a difficult and costly endeavor for the traditional medication research and development process. Identification of new targets for therapy in drug discovery must be accelerated by using the integration of genomics [13]. Combining genome-wide association study (GWAS) data with molecular quantitative trait locus (molQTL) data, such as gene expression quantitative trait locus(eQTL) or protein quantitative trait locus(pQTL), enables identifying target genes linked to risk variants via causal inference [14]. Using MR to study if an exposure causes an outcome, imitating a randomized trial with genetic data, sidestepping the need for drug testing [15]. MR analyses have been applied on multiple neurological diseases, such as Parkinson’s disease(PD) [16] and Alzheimer’s disease(AD) [17].

In this study, we selected instrumental variables (IVs) linked to eQTLs and pQTLs, which allows direct causal inferences about gene expression or protein levels for migraine. Since MR alone might not be adequate for pinpointing reliable proteins within causal pathways, subsequent colocalization, SMR, HEIDI test, and LDSC were conducted. Finally, a pharmacological evaluation was done to see if it could be a treatment for migraine.

## Methods

### Study design

**Fig. 1:**
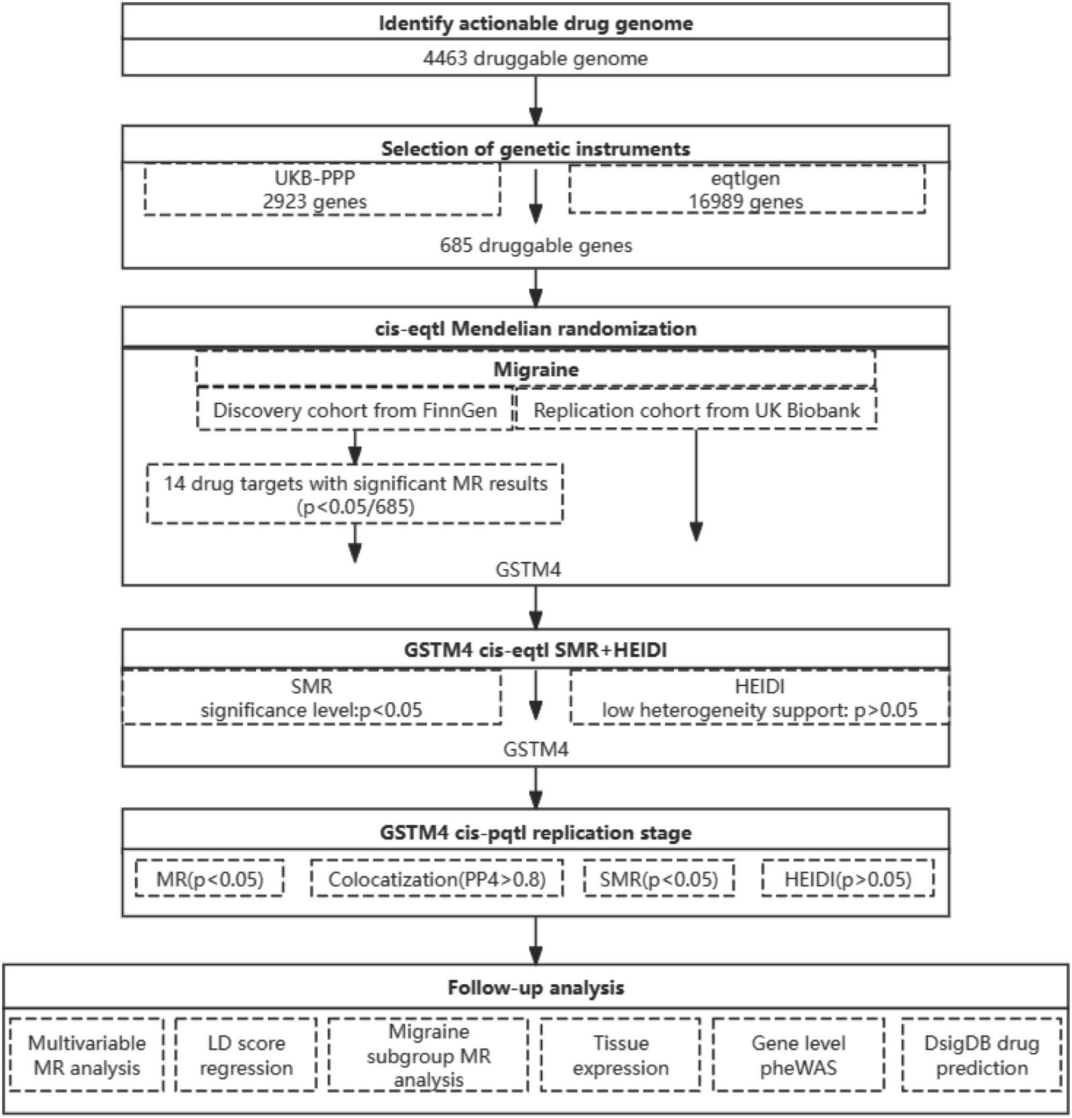
Overview of the study design

Figure 1 illustrates the analysis process. First, we took 4463 druggable genes out of a review. Next, we selected the intersection of druggable genes, pQTL genes from UKB-PPP, and eQTL genes from eQTLGen. After selecting eQTL instrumental variables, MR analysis was conducted on migraine data from FinnGen. Target druggable genes that reached significance thresholds after multiple test adjustments were validated in the UK Biobank cohort to identify potential migraine targets. We performed strict sensitivity analysis and compared the consistency of the direction of result across the replication and discovery stages. SMR analysis of the validation genes was performed at the gene expression level and further analyses at the protein level, including pQTL MR analysis, subgroup analysis, and SMR analysis. To examine if these results were impacted by distinct causative variants that were in linkage disequilibrium with one another, we also conducted colocalization and HEIDI tests. Target genes were further verified by tests for reverse causation, horizontal pleiotropy, and heterogeneity. To evaluate the combined causative effects of several risk variables, multivariate MR analysis was performed. LDSC Regression (https://github.com/bulik/ ldsc) was used to estimate the LD Score of each SNP to infer its association strength with the disease. Finally, the potential clinical applicability of the identified drug targets was evaluated by PheWAS and drug prediction.

### Exposure data

Table S1 lists the data sources. The eQTLs data, containing 16,989 genes from 31,684 blood samples of healthy European ancestors, were derived by the eQTLGen consortium(https://eqtlgen.org/). A comprehensive overview of the data is available in the original text [18].

The pQTL data were acquired from the UKB-PPP (http://ukb-ppp.gwas.eu) [19], which is a partnership between the UK Biobank (UKB) and various bio-pharmaceutical enterprises. This collaborative endeavor scrutinized plasma proteomic signatures within a cohort of 54,219 UKB participants. 2,923 proteins were conducted by comprehensive pQTLs mapping through meticulous analysis, uncovering a total of 14,287 significant genetic associations.

The compilation of druggable genes utilized in this study was derived from a previous investigation employing a computational methodology that integrated numerous existing GWAS data. This computational approach aimed to find druggable proteins and link them to well-known pharmaceutical agents, resulting in the identification of 4463 druggable genes [20]. Chris et al. focused on genes located on autosomal chromosomes and annotated by the HGNC for further analysis. This subset included 2360 genes from well-established drug target families, 674 genes related to proteins targeted by licensed medicines or compounds, and 1425 genes encoding protein targets currently in clinical development.

### Outcome data

#### Migraine

Two different datasets were used for the outcome data. The discovery cohort dataset was obtained from FinnGen Release 10 [21], published in December 2023, accessible at https://www.finngen.fi/en. In this dataset, migraine cases totaled 44,616, while 367,565 individuals were classified as healthy. Moreover, the replication cohort dataset was sourced from UK Biobank [22], including 26,052 cases and 487,214 controls. This comprehensive approach robustly validates our findings between different datasets. Furthermore, the outcome data utilized for subgroup analysis, including data on MA and MO, were also extracted from FinnGen Release 10 database, contributing to the depth and specificity of our investigation.

#### Risk factors

We identified 9 migraine risk factors encompassing epilepsy, hypertension, anxiety, insomnia, blood calcium levels, blood magnesium levels, insulin resistance, depression, and stoke (Table S2). In accordance with the original GWAS protocols, all individuals gave their informed consents. Furthermore, all ethical clearances pertinent of the GWAS were secured by the original authors of the GWAS.

### Statistics

#### Mendelian randomization analysis

MR analysis was conducted by the “TwoSampleMR” R package(version 0.5.7) [23]. Prior to MR testing, stringent quality control was performed on the SNP instruments. We selected strong instrumental variants with F-statistic ≥ 10(F-statistic= (beta/se)2) [24]. Then, based on the 1000 Genomes European panel, variations with low linkage disequilibrium (LD r2 < 0.1) [25] were carefully chosen. Finally, genes where SNPs accounted for a larger percentage of outcome variance than exposure were eliminated by Steiger filtering [26].

For genes with several instruments in the main analysis, meta-analyzed using the IVW, weighted median and MR-Egger methods were used to SNP estimations. Wald ratio estimates were calculated for each SNP. The IVW method provides the most statistical power under the assumption that all instruments are valid [27]. When the results of the three MR methods were inconsistent, the IVW was used as the primary result [28]. In contrast, the weighted median MR can tolerate up to 50% invalid instruments by weighting and taking the median of the SNP-specific estimates, with bootstrapped standard errors [29]. This method is robust to horizontal pleiotropy, a phenomenon that certain SNPs affect the result through different paths. Horizontal pleiotropy is also assessed by MR-Egger, although its statistical power is less than that of IVW [30]. It suggests that pleiotropy will not significantly bias the IVW results when all three approaches produce consistent estimates. Cochran’s Q and MR-Egger intercept tests were employed to evaluate horizontal pleiotropy and heterogeneity, respectively, for genes containing more than two instruments [31].

Sensitivity analyses for multiple testing were performed with the use of Bonferroni correction for the adjusted significance threshold. In the FinnGen discovery cohort, *p*<7.30e-5(*p*<0.05/685) was considered significant. These significant genes underwent quality control, verifying consistency across MR methods and checking for horizontal pleiotropy using MR-Egger. Because SNPs of two genes (SERPINI1 and LTB) were not found in the replication cohort, genes that passed above tests were replicated at a significance threshold of *p* < 0.0042 (*p* < 0.05/12) in the UK Biobank cohort. When the results of the three MR methods were inconsistent, the IVW method was prioritized as the primary result, and the genes with the same direction of effect in the IVW results between the replication and discovery phases were selected as the candidate drug targets. This rigorous replication approach enhanced the robustness of the findings by reducing false positives and providing external validation. Furthermore, relationships between GSTM4 and the risks of migraine were examined by using the “MVMR” R package (version 0.4), taking into account the other variable. MVMR can identify the combined causative impact of multiple risk variables [32].

#### Reverse causality detection

We carefully selected genetic instruments for migraine from the FinnGen dataset, adhering to the same criteria used for eQTLs. To investigate potential reverse causation, these instruments were then employed in a bidirectional MR analysis [26]. To ensure the validity and reliability of the observed connections, we established a strict criterion and the weighted median, MR-Egger, and IVW methods were used to calculate the effect estimates at a threshold of *p* < 0.05. Additionally, the directionality of the link between pQTLs and migraine was verified using Steiger filtering.

### Colocalization analysis

Colocalization analysis was carried out using the “coloc” R package (version 5.2.3) for genes with MR correlations significantly across cohorts [33]. It was determined if the gene expression-migraine association was due to common causal variants by Bayesian method. Five hypotheses were tested [34]: H0: no association, H1: association with expression only, H2: association with migraine only, H3: independent associations, and H4:shared causal variant. For trait-specific relationships, prior probability were set at 1e-4, and for shared variants, at 1e-5. A posterior probability (PPH4) ≥ 0.8 was considered as a strong colocalization, and genes with strong colocalization were considered as potential drug target candidate genes. Visualization of the regional results was performed using the “LocusCompareR” package(version 1.0.0) [35]. The STROBE-MR checklist is provided as Table S3 [36], and the study was carried out in accordance with the current guideline.

### SMR analysis and HEIDI test

Compared with most other methods for an integrative analysis of GWAS and eQTL data, the SMR and HEIDI approach features the ability to distinguish a pleiotropic model from a linkage model [37]. SMR analysis was carried out as a supplementary method to confirm the causal relationships between migraine and gene expression using the SMR software (version 1.3.1) [38]. The HEIDI test was used to demonstrate that proteins associated with migraine was not due to genetic linkage [38]. A significant SMR association was defined as *p* < 0.05, while HEIDI *p* > 0.05 indicated that the association was caused by a shared genetic variant. This additional analysis provided further support for the causal relationships identified through the primary Mendelian randomization approach.

### LDSC analysis

To evaluate the genetic association between GSTM4 and migraine, LDSC (https://github.com/bulik/ldsc) [39] was employed, which ranges from − 1 to 1. A complete negative genetic correlation is represented by a value of -1 in the estimate, while a complete positive genetic correlation is represented by a value of 1. In order to study the inflation effect resulting from a polygenic signal or bias, LDSC investigates the correlation between test statistics and linkage disequilibrium. This approach is not influenced by sample overlap and can evaluate genetic association using GWAS data.

### Expression in different tissue

To analyze the expression of GSTM4 in human tissues, we used the Human Protein Atlas (https://www.proteinatlas.org) [40], which displays the mRNA and protein levels of gene in human tissues. The protein expression data, from 44 normal human tissues, cover 15,323 genes (76%). Exploring the expression of GSTM4 in different tissues and different brain regions can suggest its possible mechanism as a therapeutic target for migraine.

### Phenome-wide association analysis

Based the AstraZeneca PheWAS Portal (https://azphewas.com/) and the PheWeb database (https://pheweb.org/), a PheWAS was performed to evaluate the pleiotropic effects of potential therapeutic targets and possible adverse effects [41]. Data from about 15,500 binary and 1,500 continuous phenotypes were utilized in the original study. The individuals in the exome sequencing subgroup were taken from the UK Biobank. The original publication contains a description of the comprehensive technique [42]. This comprehensive PheWAS analysis provides insights for understanding the complicated traits at genetic basis and evaluating the safety and efficacy of drug targets.

### Candidate drug prediction

The target gene was submitted to the Drug Signatures Database (DSigDB, http://dsigdb.tanlab.org/DSigDBv1.0/) [43] to assess potential protein-drug interactions. DSigDB is an extensive database that makes it easier to identify connections between drugs, chemicals, and the target genes. It has 22,527 gene sets and 17,389 unique compounds linked to 19,531 genes. This assessment of interactions between drugs and proteins is crucial to determine if the discovered genes can be effectively used as therapeutic targets. Specifically, the target genes were uploaded to the Enrichr suite of gene set enrichment analysis tools (https://maayanlab.cloud/modEnrichr/) [44] to leverage the DSigDB database and predict potential drug candidates that may target the gene of interest.

## Results

### Selection of genetic instrument variants

**Fig. 2:**
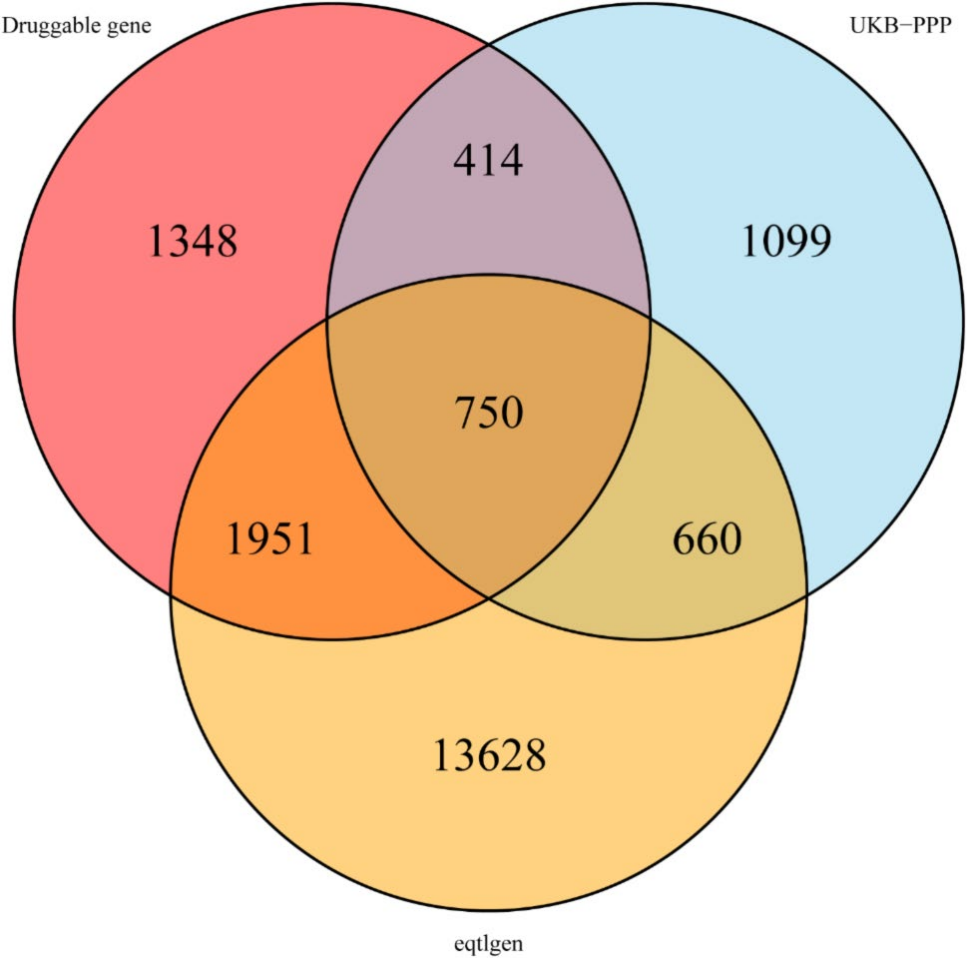
Venn diagram of three datasets

We identified 2701 druggable genes by intersecting the druggable genes with the significant cis-eQTL from the eQTLGen Consortium. To verify results by UKB-PPP cis-pQTL data, we intersected these genes and eventually obtained 750 druggable genes (Fig. 2). Based on the selection criterion of instrument variants, we identified 3073 cis-eQTL for 685 druggable genes as IVs after clumping in discovery analysis.

MR analysis in discovery phase between gene expression and migraine.

**Fig. 3:**
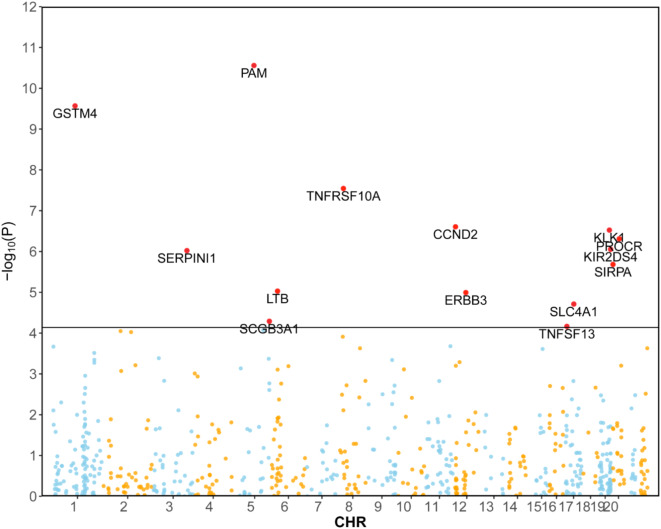
Manhattan plot for correlation of druggable genes with migraine in MR analysis

**Fig. 4:**
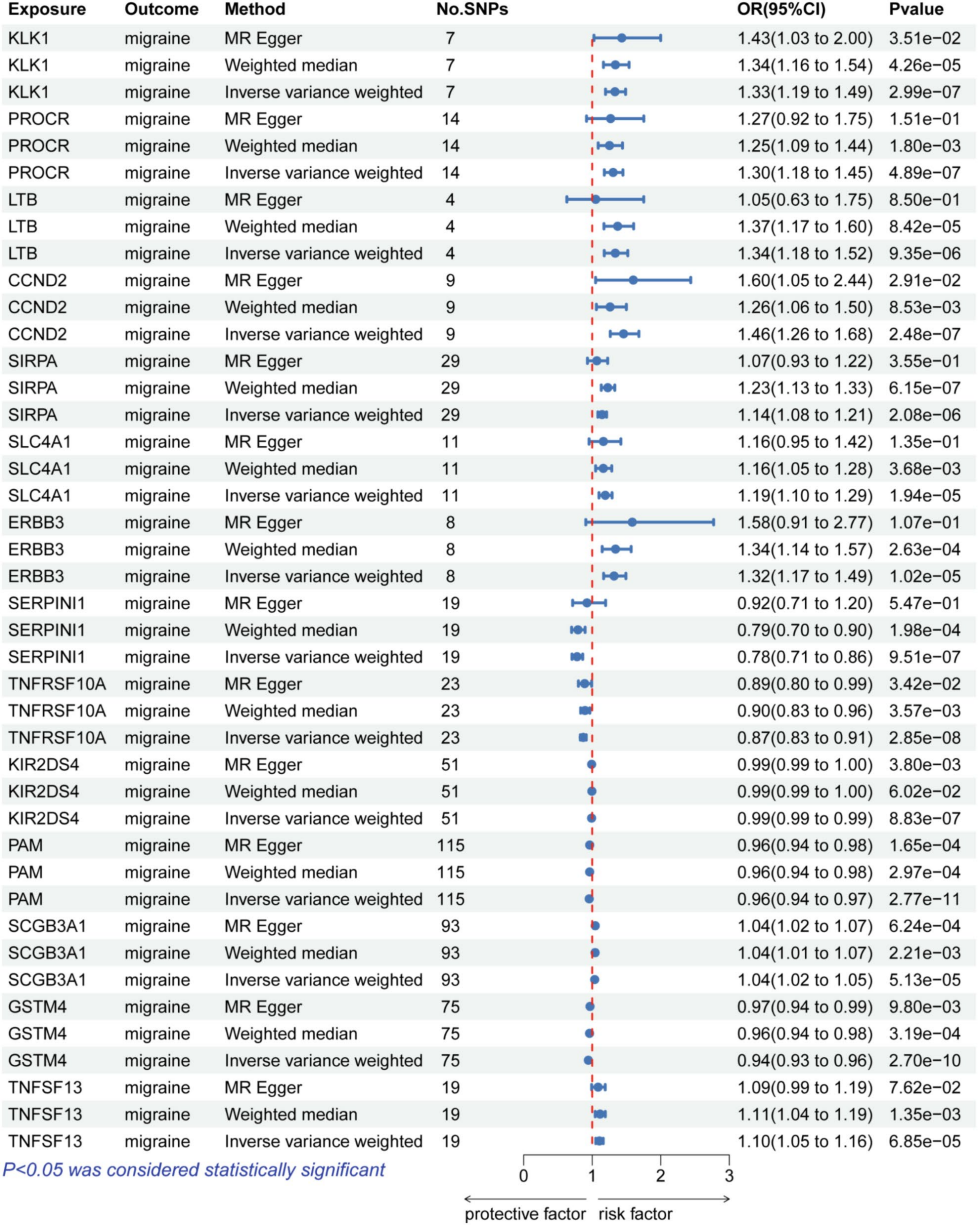
Forest plots illustrating the results of the discovery stage for 14 essential genes

In the discovery cohort, we performed a two-sample MR analysis on migraine patients, including 44,616 cases and 367,565 controls from the FinnGen cohort. Table S4 displayed the genetic variations of eQTLs that were employed in the discovery phase. At Bonferroni significance (*P* < 7.30e-05, IVW or Wald ratio), 14 genes were significantly related to the risk of migraine (Figs. 3 and 4). These genes include KLK1, PROCR, LTB, CCND2, SIRPA, SLC4A1, ERBB3, SERPINI1, TNFRSF10A, KIR2DS4, PAM, SCGB3A1, GSTM4 and TNFSF13. In the primary analysis, no heterogeneity (*P* > 0.05, Table S5) or horizontal pleiotropy (*P* > 0.05, Table S6) was found, and all 14 genes demonstrated a consistent direction of impact across the three methods (Table S7). The Steiger filtering further confirmed the directionality of the connection between gene expression and illness state(Tables S8).

### MR analysis in replication phase between 14 genes and migraine

**Fig. 5:**
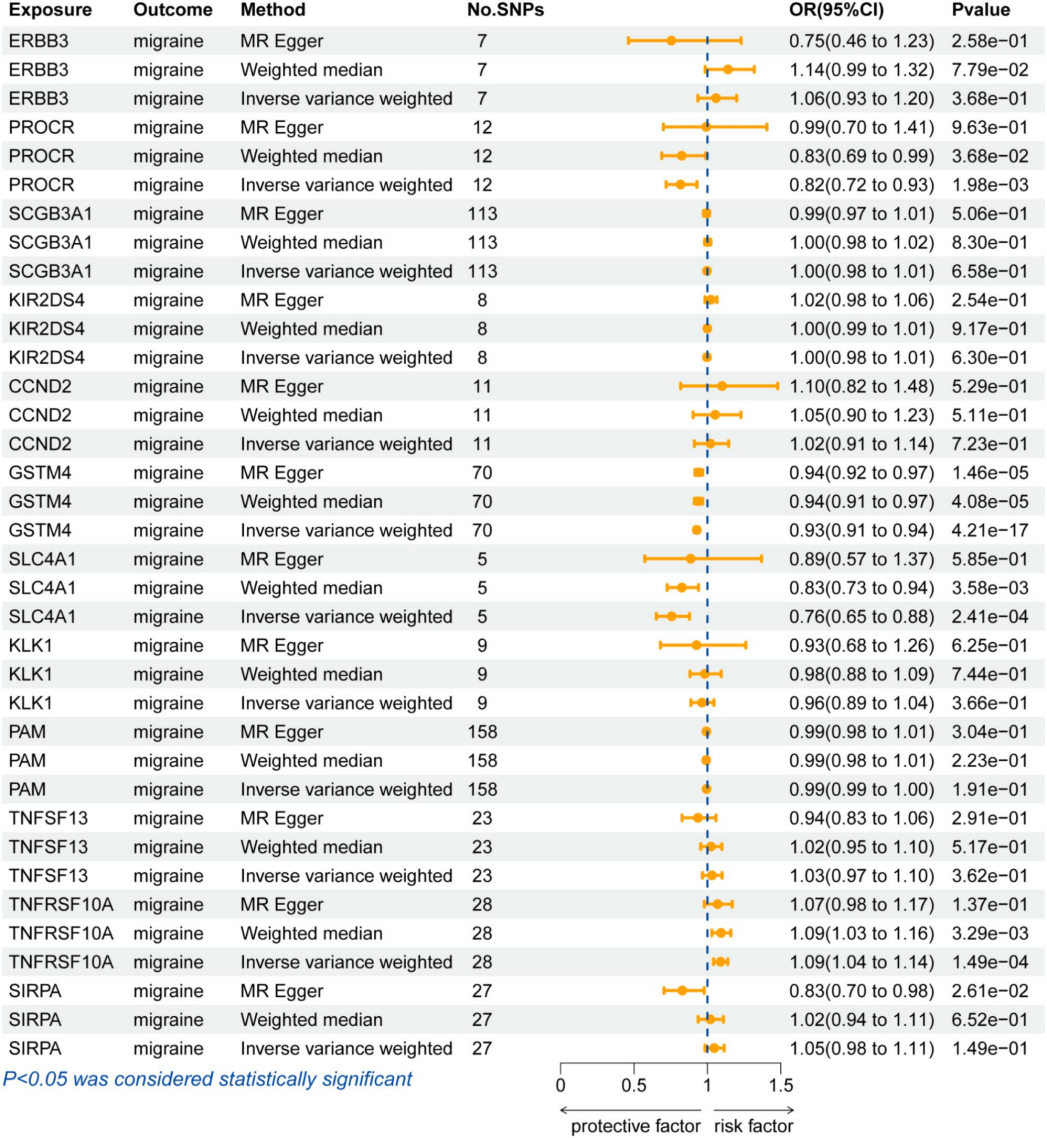
Forest plots illustrating the results of the replication stage for 12 genes

Due to the lack of corresponding SNPS, only 12 genes identified in the discovery phase were subjected to MR Analysis using data from the UK Biobank cohort to replicate the results. The results showed that a reduced risk of migraine was related with PROCR, GSTM4, and SLC4A1(PROCR: OR(95%CI) = 0.82(0.72–0.93);*p =* 1.98e − 03;GSTM4:OR(95%CI) = 0.93(0.91–0.94);*p =* 4.21e − 17;SLC4A1:OR(95%CI) = 0.76(0.65–0.88); *p =* 2.41e − 04), while TNFRSF10A was related with increased migraine risk (TNFRSF10A: OR(95%CI) = 1.09(1.04–1.14); *p =* 1.49e − 04) (Fig. 5 and Table S9). In the sensitivity analysis, there was no heterogeneity (Table S10) and horizontal pleiotropy (Table S11) in all 4 gene. Steiger filtering was also verified (Table S12). However, except for GSTM4, the remaining three genes were excluded due to inconsistent directionality of impact in both the discovery and replication phases.

### SMR and HEIDI test between GSTM4 in gene expression and migraine

**Fig. 6:**
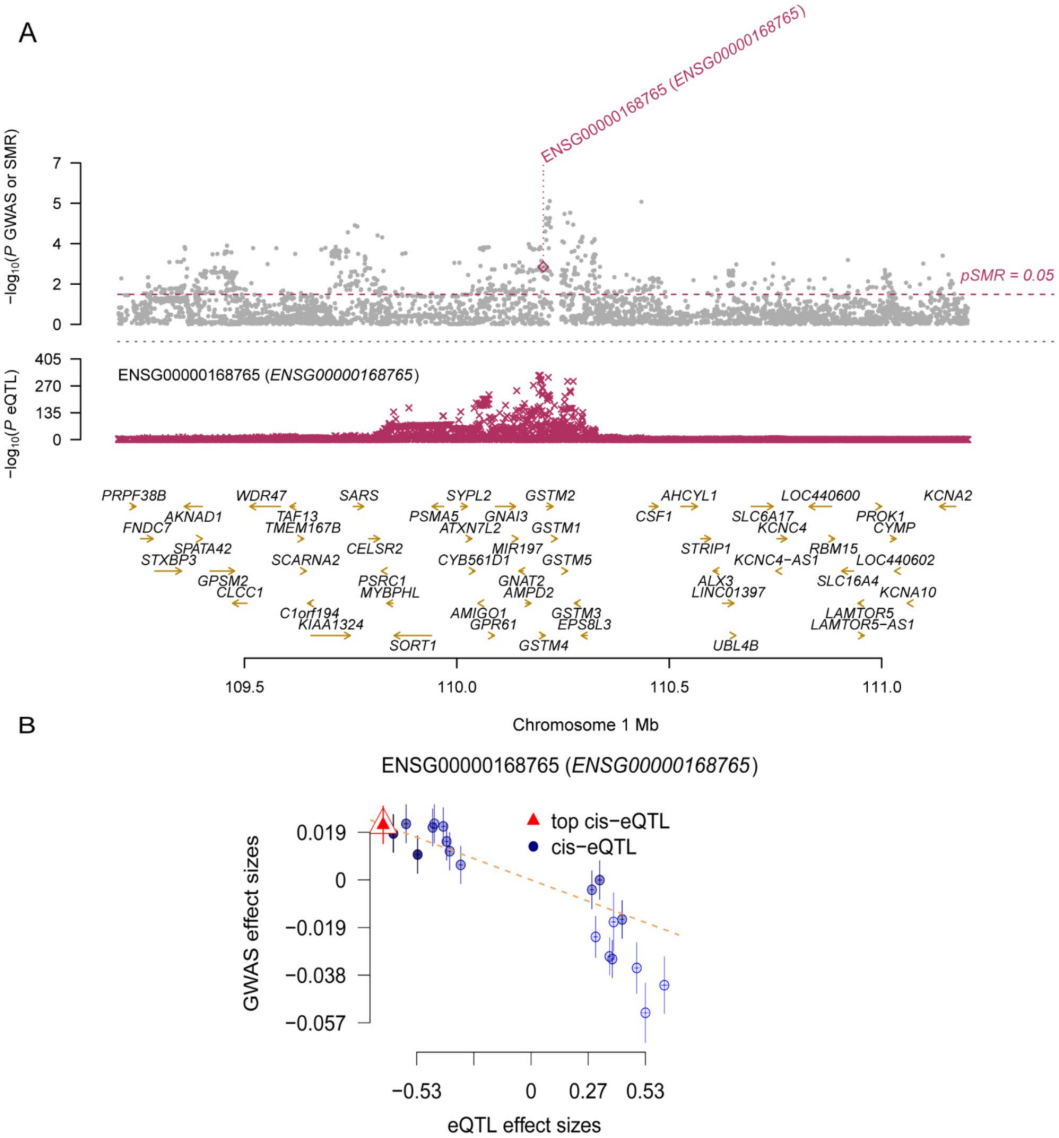
The SMR locus plots and effects plots for correlations of GSTM4 in gene expression with migraine

In order to further eliminate and the effect of pleiotropy and linkage disequilibrium, we conducted SMR and HEIDI test in order to confirm the above results for the GSTM4 cis-eQTL. It completely agreed with the MR results in the direction of effect and passed both the HEIDI test (*p* > 0.05) and the SMR test (*p* < 0.05) (Table S13). The plots of SMR locus and effect are shown in Fig. 6A, B.

### MR and subgroup analysis between GSTM4 in protein level and migraine

**Fig. 7:**
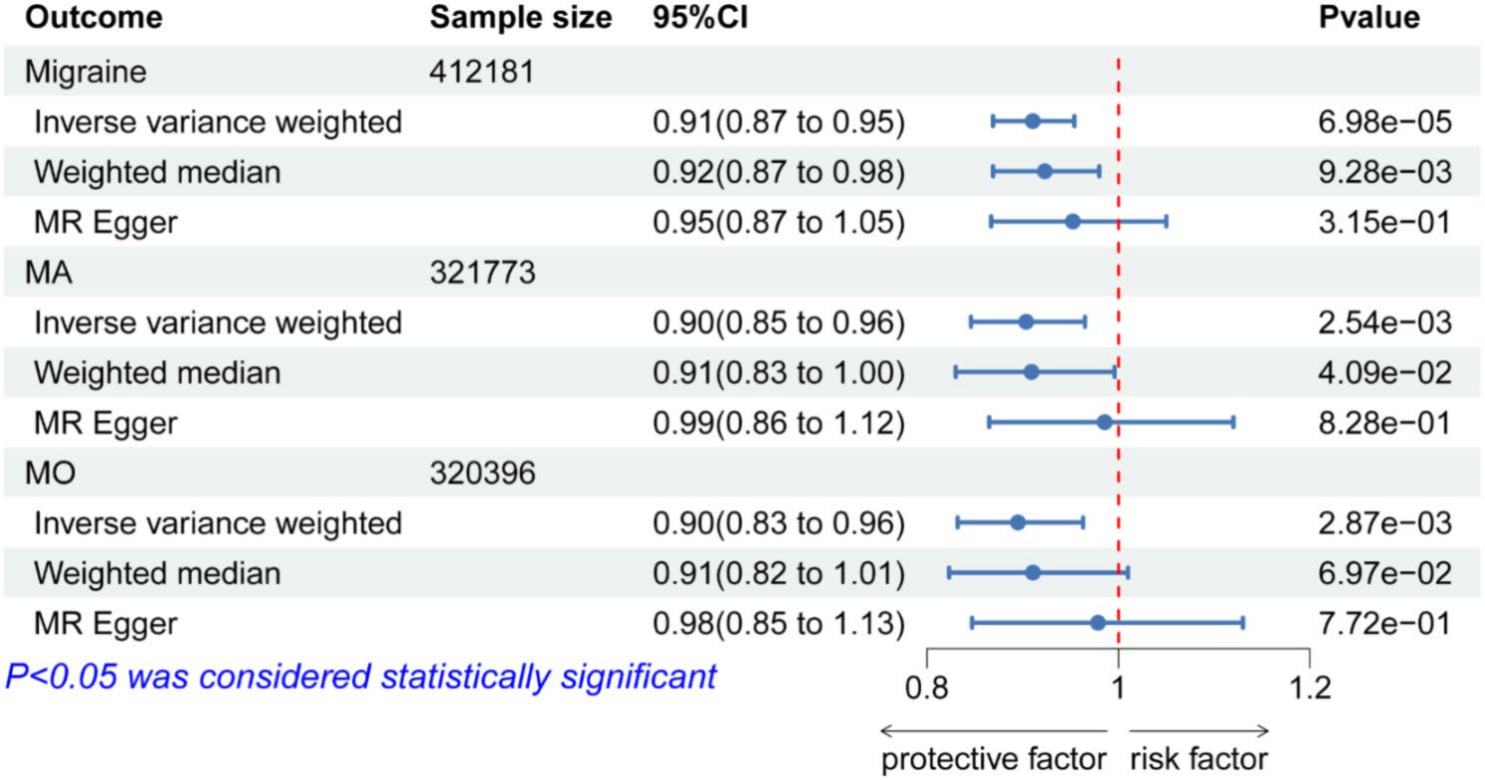
Forest plots illustrating the results of circulating GSTM4 and subgroup analysis

To further determine the possibility of GSTM4 as a migraine treatment target, we used cis-pQTL data of GSTM4 from UKB-PPP for MR analysis. In the primary analysis, it revealed significant associations between circulating GSTM4 and migraine(instruments of GSTM4 cis-pQTL in Table S14; MR results in Table S15). The increased SD of circulating GSTM4 predicted by genes was found to be connected with a lower incidence of migraine (OR(95%CI) = 0.91(0.87–0.95); *p =* 6.98e-05), demonstrating convincing evidence of a correlation with the risk of migraine(Fig. 7).

In the sensitivity analysis, no heterogeneity (Table S16) or horizontal pleiotropy (Table S17) was observed across GSTM4. The reverse MR analysis did not show any causal effect of migraine on GSTM4 levels(Table S18), and the results of Steiger filtering ensured the directionality of effect (Table S19). Additionally, subgroup analyses further suggested a causal relationship between GSTM4 and MA or MO (Fig. 7). The increased protein level of genetically predicted GSTM4 was linked to lower risks of MA(OR(95% CI) = 0.90(0.85–0.96);*p* = 2.54e-03), as well as lower risks for MO(OR(95% CI) = 0.90(0.83–0.96); *p* = 2.87e-03).

### Colocalization analysis between GSTM4 cis-pQTL and migraine

The gene GSTM4 consistently exhibited negative estimated effects in both cis-eQTL and cis-pQTL MR analyses, indicating a correlation between increased GSTM4 expression and decreased migraine risk. Utilizing GSTM4 agonists could present an innovative and reliable approach to mitigate migraine risk.

**Fig. 8:**
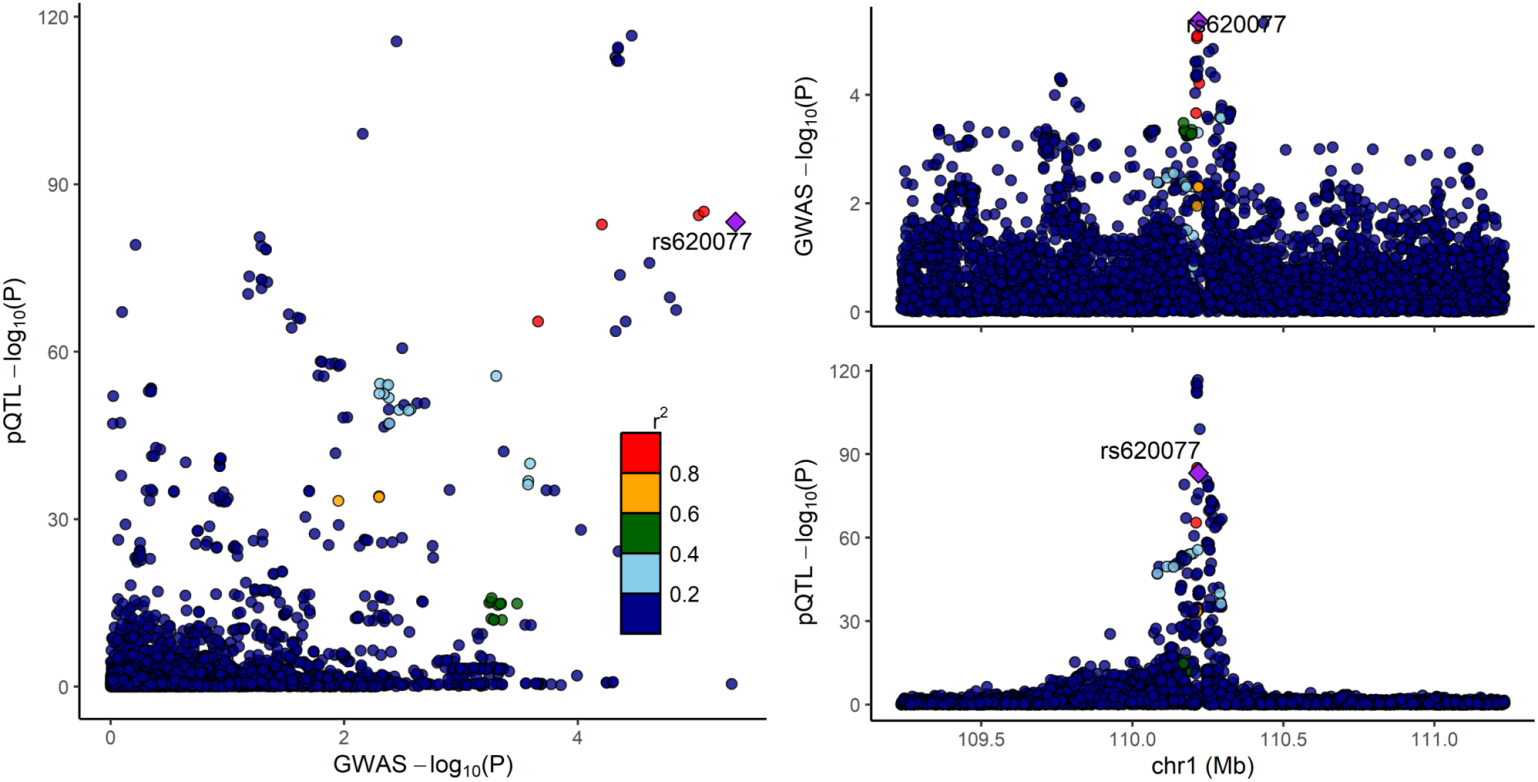
Regional plot of colocalization evidence of GSTM4 and migraine

With a high degree of confidence that the causal variation underlying the relationship between the protein level of GSTM4 and migraine risk is shared, the colocalization analysis (PPH4 = 0.86) suggested that linkage disequilibrium was not a contributing factor to the observed MR findings for this gene(Table S20, Fig. 8).

### SMR and HEIDI tests verified GSTM4 cis-pQTL

**Fig. 9:**
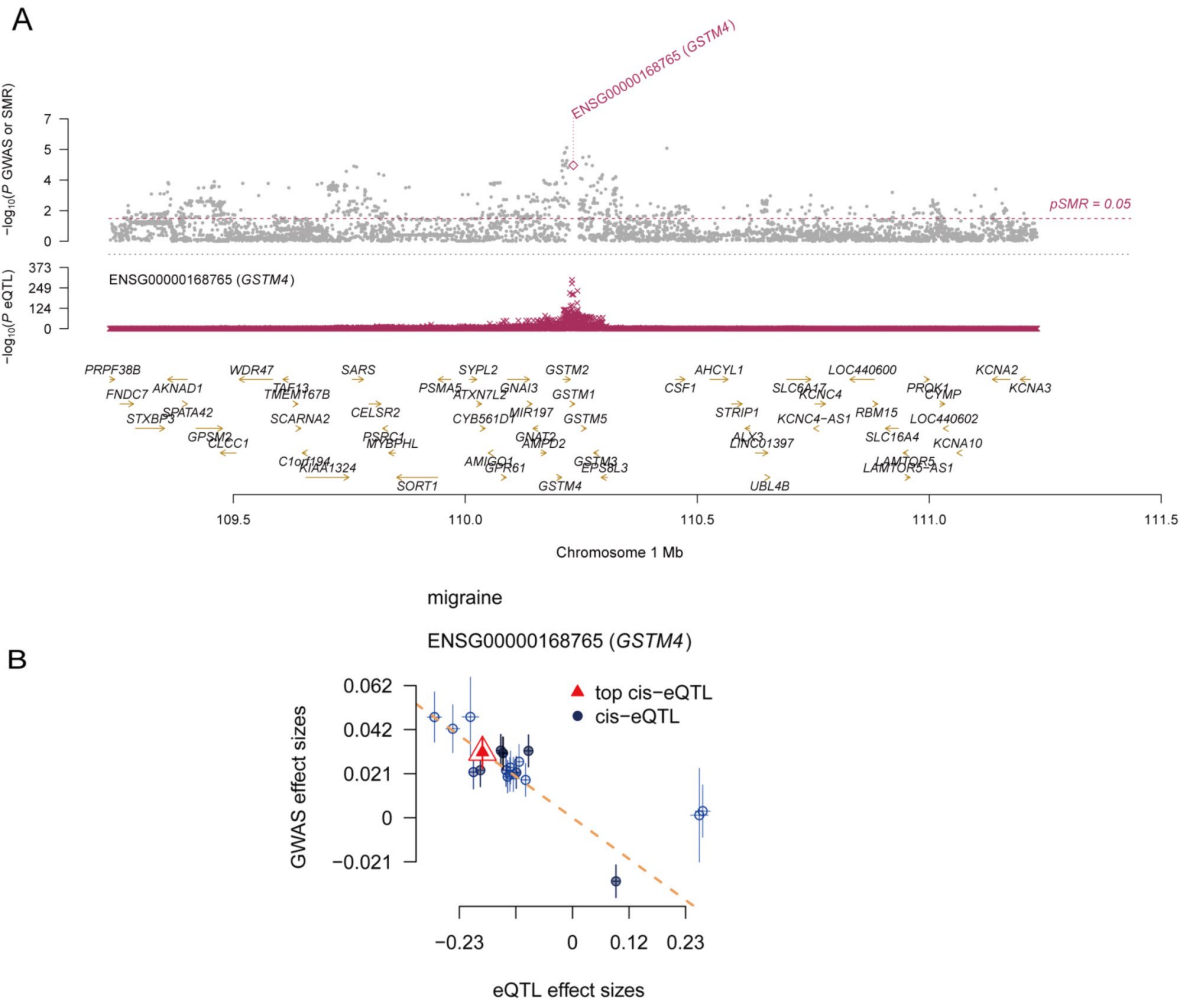
The SMR locus plots and effects plots for correlations of circulating GSTM4 with migraine

GSTM4 cis-pQTL also passed the SMR test (*P* < 0.05) and the HEIDI test (*p* > 0.05) (Table S21, Fig. 9A, B). Taken together with the above evidence, we conclude that GSTM4 may be a promising drug target for migraine.

### MVMR analysis identified GSTM4 was independently associated with migraine

**Fig. 10:**
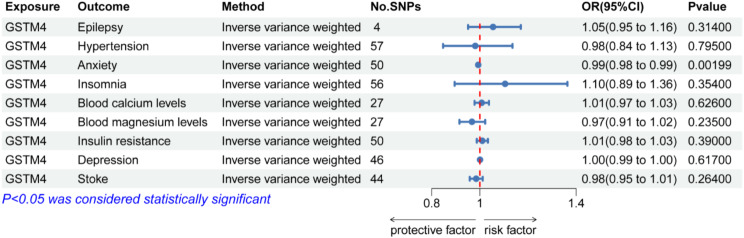
Associations between GSTM4 and migraine risk factors

Among the 9 risk factors for migraine, GSTM4 was significantly associated with anxiety (*p =* 0.002 [IWW], Fig. 10). To test the independent association between GSTM4 and migraine, we performed a MVMR analysis to reveal a significant independent correlation between GSTM4 and migraine(*p =* 0.001 [IWW], Table S22).

### GSTM4 expression in different tissue

**Fig. 11:**
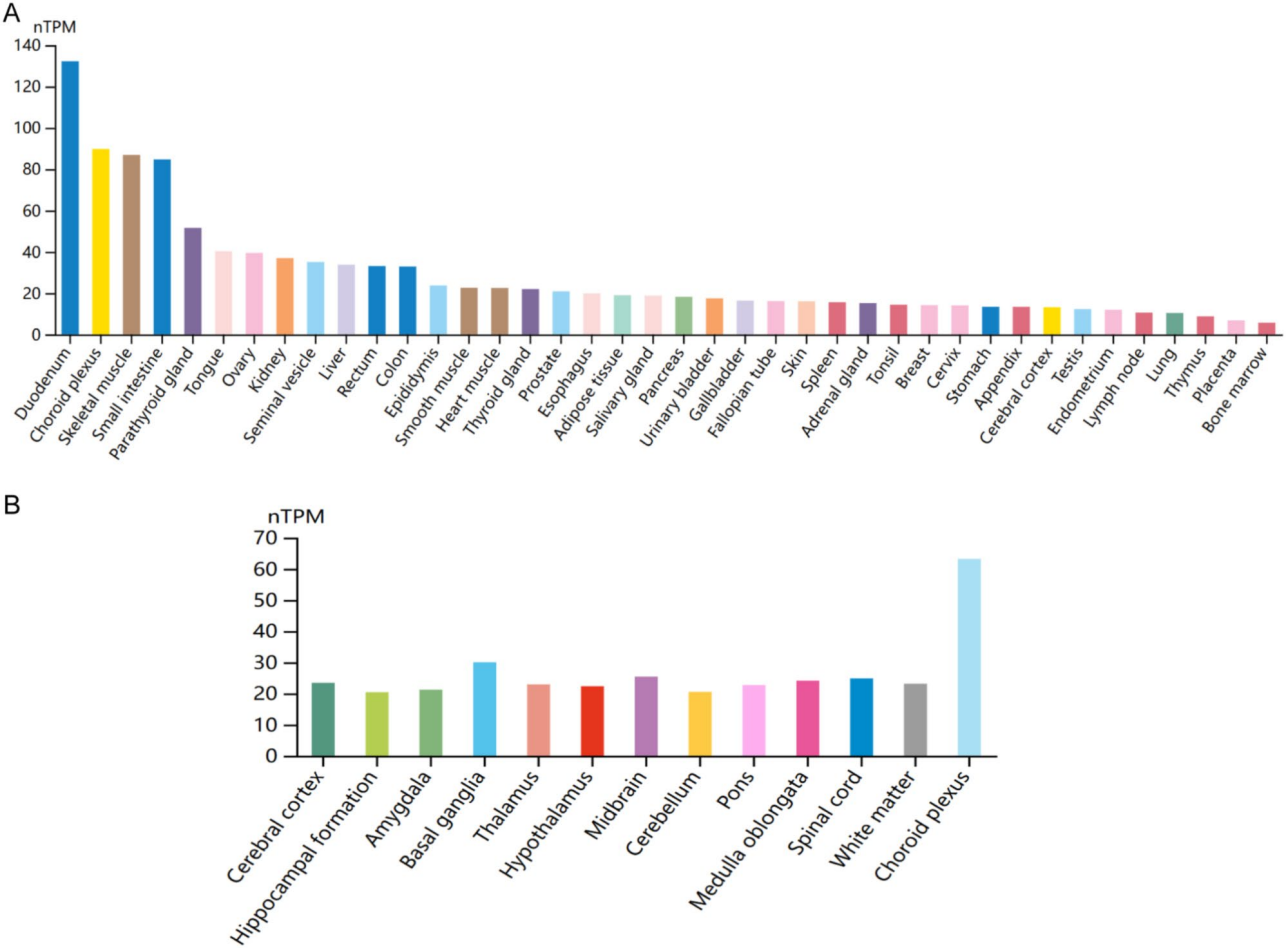
The expression of GSTM4 in human tissues and brain regions

We identified the differences in the expression of GSTM4 in human tissues and brain regions through Human Protein Atlas. The results showed that GSTM4 was mainly expressed in small intestine and choroid plexus (Fig. 11A, B). This may provide explanation for the target tissue and pathway of GSTM4.

### LDSC analysis

To assess the genetic correlation, we performed LDSC analysis between GSTM4 and migraine. The results showed a significantly negative genetic correlation for GSTM4 with migraine (rg= -0.1977, *p =* 0.0145). Given the negligible sample overlap between the GSTM4 and migraine datasets, we further constrained the intercept of the genetic covariance estimate to zero. By doing so, LDSC gains greater statistical power with slightly reduced standard errors. Consequently, an even more significant negative genetic connection was found between GSTM4 and migraine (rg = -0.4228, *p =* 0.0004). Detailed information is showed in Table 1.

**Table 1:** Genome-wide genetic correlations between GSTM4 and migraine using constrained and unconstrained LDSC

		Unconstrained LDSC			Constrained LDSC		
Trait1	Trait2	r_g_	se	p	r_g_	se	p
GSTM4	Migraine	-0.1977	0.0809	0.0145	-0.4228	0.1189	0.0004
r_g_, genetic correlation; se, standard error.							

### PheWAS

**Fig. 12:**
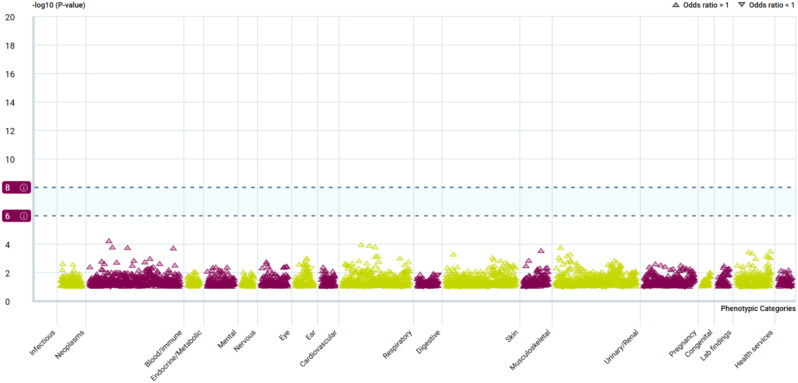
Binary traits PheWAS association with GSTM4

Using the PheWAS Portal and PheWeb database, we conducted phenome-wide MR to determine the possible side effects of targeting GSTM4. The results found no evidence of a significant relationship between GSTM4 and other phenotypes in the PheWeb database (Tables S23) or the PheWAS Portal (Fig. 12) at the genome-wide significance level (*p* < 5e-08). These results reinforce the validity of our findings and suggest a low risk of adverse drug reactions or unintended horizontal pleiotropic effects if GSTM4 was to be targeted therapeutically.

### Candidate drug prediction

Evaluating GSTM4 as a possible drug target requires an assessment of the protein-drug interaction. To find possible agents, GSTM4 was examined using the DSigDB drug database on Enrichr. According to the results, the top four drugs associated with GSTM4 are VITAMIN E(CTD 00006994), Sodium salicylate(CTD 00006761), Hydralazine (CTD 00006108), and Tesaglitazar(CTD 00004468) (Table 2).

**Table 2:** Candidate drug predicted using DSigDB

Term	*P* value	Adjusted *P* value	Odds ratio	Combined score	Genes
Sodium salicylate	< 0.001	0.017	36.17	323.68	GSTM4
Tesaglitazar	0.002	0.097	33.82	207.50	GSTM4
VITAMIN E	< 0.001	< 0.001	3.70	50.62	GSTM4
Hydralazine	< 0.001	0.033	3.42	26.02	GSTM4

## Discussion

Migraine affects the life quality of patients significantly. Despite recent advances, current migraine therapies remain comparatively disappointing, as they fall short of fully meeting clinical needs. Thus, it is very crucial to discover new drugs for migraine.

Our study found an association between increased GSTM4 expression and decreased migraine risk, which was also observed in MA and MO. The results emphasize the possibility of GSTM4 agonist as a therapeutic intervention. Other analysis ensured the reliability of the results.

The possible mechanisms of migraine include hypothalamic and brainstem activation, cortical spreading depression(CSD) and trigeminovascular activation [45]. The role of oxidative stress in migraine is supported by studies that reported the decreased serum total antioxidant status and increased oxidative stress index (OSI) of migraine patients [46–49].Certain triggers of migraine including internal and external stimuli can increase oxidative stress, such as hormonal changes [50], psychological stress [51], lack of sleep [52], and intense sensory stimulation [53], so as to activate the metabolic changes in the brain. The hypothalamus plays an important role in the early stages of migraine [54]. It can sense the changes in the brain metabolism [55]. Oxidative stress reduces cerebral ATP and glycogen levels and increases cerebral excitability. The process can affect CSD susceptibility, an electrophysiological phenomenon resulting in migraine with aura [56]. Reactive oxygen species (ROS) generated in oxidative stress are involved in the coupling between CSD and activation and/or sensitization of the trigeminal neurovascular injury sensory system [57]. However, most patients with migraine never experience an aura. Thus, metabolic changes may directly activate the trigeminovascular system. A study showed that KATP channels link metabolic stress with activation of trigeminovascular nociceptors [58].

Glutathione S-transferase (GST) is believed to play a role in providing protection against oxidative stress and toxic foreign chemicals by catalyzing their conjugation to glutathione [59]. Previous study found that the pathophysiology of asthma in children may include the GST-T1 null genotype and elevated oxidative stress, which suggested that GST activity is tightly associated with oxidative stress [60]. Meanwhile, in migraine patients, serum/plasma GST activity has been found decreased [61]. The degree of GST gene polymorphism can alter the sensitivity to medical treatments of migraine and susceptibility to migraine [62].

Currently, six membrane-bound GSTs and eight different classes of soluble GSTs (alpha, kappa, mu, omega, sigma, theta, pi, and zeta) have been discovered. The GST enzyme GSTM4 is a member of the mu class. While GSTM4 and other GSTM enzymes have a significant degree of amino-acid sequence similarity, they have different physiochemical characteristics and tissue distributions [63].

It has been demonstrated that patients having lower GSTM4 enzyme expression may be less effective in eliminating oxidatively damaged molecules and therefore be more susceptible to the consequences of radical oxygen species attack [64]. Thus, the GSTM4 may influence the development and progression of migraine through regulating the oxidative stress-related pathways. Additionally, the previous study has found that GSTM4 expression level affects the oncogenic and drug-resistant properties of Ewing’s sarcoma cells [65] provides a relevant precedent for investigating the potential of GSTM4 as a therapeutic target in migraine.

In summary, the available evidence supports the notion that both oxidative stress and the GST family member GSTM4 play an important role in the potential treatment and pathogenesis of migraine. These findings call for further in-depth researches to elucidate the precise mechanisms by which GSTM4 may contribute to migraine development and its potential as a therapeutic target.

Previous research has demonstrated a connection between migraine and the elimination of metabolic waste from the gut-brain axis and cerebrospinal fluid [66, 67]. A study showed that the activity of GST expression in the intestine of rats with the knockout of LanCL1 gene, which has a protective effect on oxidative stress in the brain, was reduced, indicating that GST plays a role in the gut-brain axis against oxidative stress [68]. Our study also found that GSTM4 is mainly distributed in the small intestine and choroid. On the one hand, the small intestine, as an important link of the gut-brain axis, is closely related to nervous system diseases; On the other hand, the choroid is an important link of cerebrospinal fluid circulation and is closely related to the removal of cerebral metabolic waste. This further provides evidence that GSTM4 plays a role in the gut-brain axis. Therefore, we speculate that the decrease of GSTM4 expression causes the disruption of gut-brain axis and leads to migraine.

The PheWAS suggested the small potential side effects of GSTM4, significantly lowing the possibility of pleiotropy-related bias. Given the individualization of migraine patients and comorbidities, one goal of migraine treatment is to minimize treatment-related side effects in each patient’s clinical course [69]. This comprehensive evaluation strengthens the evidence supporting the druggable potential of the target gene, which is crucial given the challenges of drug side effects and inconclusive clinical trial results.

There are numerous strengths in this study, including two separate migraine population data, mutual validation in the gene expression and protein level, supportive HEIDI test and colocalization analysis, LDSC, and the elimination of horizontal pleiotropy through MVMR analyses and PheWAS.

It is important to take limitations into account when evaluating our results. First, the fact that only individuals of European ancestry were included in our analysis means that the findings could not be applied to other ethnic ancestries. Secondly, when conducting the multifactorial MR analysis, we only selected a portion of risk factors for migraine. To lessen the possibility of pleiotropic effects, future study should concentrate on additional possible risk factors. Third, MR analysis offers insightful information on potential causal correlations, but its assumptions might not be entirely compatible with actual clinical trial conditions. MR typically assumes low-dose chronic exposure and linear dose-response, which may not reflect the short-term, high-dose treatments often evaluated in practice. Consequently, neither the MR results nor the MR-estimated effect sizes will always accurately reflect those observed under conventional clinical trials. This discrepancy is an important consideration when interpreting and applying MR findings. In addition, as a neurological disease, we attempted validation using eQTL data from brain tissue and CSF samples. However, we did not include this part of the study because of limited available data. Finally, because there are absences in the GWAS data for these subgroups, we are unable to conduct an analysis between GSTM4 and episodic or chronic migraine.

In summary, this combination of different levels of Mendelian randomization and SMR analysis identified GSTM4 agonists as potentially effective treatment targets for migraine. However, randomized controlled trials are critical to ultimately evaluate the efficacy and safety of the potential drug target.

### Electronic supplementary material

Below is the link to the electronic supplementary material.

Supplementary Material 1

## Data Availability

The original contributions presented in the study are included in the article, further inquiries can be directed to the corresponding authors.

## Abbreviations

MR: Mendelian randomization
GST: Glutathione S-transferase
OSI: Oxidative stress index
MA: Migraine with aura
MO: Migraine without aura
pQTL: Protein quantitative trait locus
SMR: Summary-data-based Mendelian randomization
IVs: Instrumental variables
AD: Alzheimer’s disease
PD: Parkinson’s disease
pQTL: Protein quantitative trait locus
eQT: Expression quantitative trait locus
molQTL: Molecular quantitative trait locus
GWAS: Genome-wide association stud
MOH: Medication overuse headache
CSD: Cortical spreading depolarization
PheWAS: Phenome-wide association study
MVMR: Multivariate Mendelian randomization
LDSC: Linkage disequilibrium score
HEIDI: Heterogeneity in dependent instruments
